# Size and Shape Filtering of Malignant Cell Clusters within Breast Tumors Identifies Scattered Individual Epithelial Cells as the Most Valuable Histomorphological Clue in the Prognosis of Distant Metastasis Risk

**DOI:** 10.3390/cancers11101615

**Published:** 2019-10-22

**Authors:** Velicko Vranes, Nemanja Rajković, Xingyu Li, Konstantinos N. Plataniotis, Nataša Todorović Raković, Jelena Milovanović, Ksenija Kanjer, Marko Radulovic, Nebojša T. Milošević

**Affiliations:** 1 Department of Basic and Environmental Science, Instituto Tecnológico de Santo Domingo (INTEC), Santo Domingo 10602, Dominican Republic; velicko.vranes@intec.edu.do; 2 Department of Biophysics, School of Medicine, University of Belgrade, 11000 Belgrade, Serbia; nemanja.rajkovic@med.bg.ac.rs; 3 Multimedia Laboratory, The Edward S. Rogers Sr. Department of Electrical & Computer Engineering, University of Toronto, Toronto, ON M5S 3G4, Canadakostas@ece.utoronto.ca (K.N.P.); 4 Department of Experimental Oncology, Institute for Oncology and Radiology, 11000 Belgrade, Serbia; todorovicn@ncrc.ac.rs (N.T.R.); jelena.mil10@gmail.com (J.M.); marko@radulovic.net (M.R.)

**Keywords:** breast cancer, tumor budding, invasion, tumor cell dissociation, individual cell, prognosis, metastasis, image analysis, particle analysis, pan-cytokeratin, histopathology

## Abstract

Survival and life quality of breast cancer patients could be improved by more aggressive chemotherapy for those at high metastasis risk and less intense treatments for low-risk patients. Such personalized treatment cannot be currently achieved due to the insufficient reliability of metastasis risk prognosis. The purpose of this study was therefore, to identify novel histopathological prognostic markers of metastasis risk through exhaustive computational image analysis of 80 size and shape subsets of epithelial clusters in breast tumors. The group of 102 patients had a follow-up median of 12.3 years, without lymph node spread and systemic treatments. Epithelial cells were stained by the AE1/AE3 pan-cytokeratin antibody cocktail. The size and shape subsets of the stained epithelial cell clusters were defined in each image by use of the circularity and size filters and analyzed for prognostic performance. Epithelial areas with the optimal prognostic performance were uniformly small and round and could be recognized as individual epithelial cells scattered in tumor stroma. Their count achieved an area under the receiver operating characteristic curve (AUC) of 0.82, total area (AUC = 0.77), average size (AUC = 0.63), and circularity (AUC = 0.62). In conclusion, by use of computational image analysis as a hypothesis-free discovery tool, this study reveals the histomorphological marker with a high prognostic value that is simple and therefore easy to quantify by visual microscopy.

## 1. Introduction

Cancer diagnosis, prognosis, and treatment are the main challenges in oncology. The primary breast tumor is not life-threatening until the disease becomes systemic by its metastatic spread. For this reason, patients are treated with cytotoxic therapy to eliminate distant micrometastases. However, as most patients do not incur metastasis even without cytotoxic chemotherapy [1], it may be that many are unnecessarily exposed to toxic side effects of chemotherapy treatment [2]. This could be resolved by prescribing less intense treatments to those at low risk and more intense chemotherapy to those reliably prognosticated at high metastasis risk.

Clinicopathological parameters with prognostic value include tumor size, lymph node spread and metastasis (TNM staging) age, histologic grade, steroid receptor status [3], and gene signatures such as Mammaprint and OncotypeDX [4]. TNM staging is accepted for disease outcome prognosis and guidance for cancer treatment [5]. However, for early breast cancer patients with negative lymph node spread and distant metastasis, this staging system is not anymore prognostically reliable because it only contains information on tumor size. New prognostic markers are therefore particularly needed in patients with N0M0 disease in order to compensate for the reduced prognostic value of TNM as the major prognostic marker.

Histologic grade has been used since the 1920s and includes tubule formation, nuclear pleomorphism, cell growth structures, and mitotic cells as morphological features capable to prognosticate breast cancer aggressiveness [6]. However, similarly to TNM, the histologic grade is prognostically less reliable in early breast cancer with most patients classified as grade 2. Furthermore, although histologic grade examines morphological features, it does not take into consideration the spatial distribution of malignant cells.

Taken together, the current prognostic methodology cannot provide sufficiently reliable risk classification, with even the most advanced gene signature tools delivering an accuracy of only 65% and an area under the receiver operating characteristic curve (AUC) of 0.69 [7]. The prognosis could be improved by the computational analysis which exploits histopathology information that cannot be quantified by microscopic inspection, such as spatial distribution, texture, shape, and complexity [8,9,10,11]. Its advantages further include high speed and cost-efficiency. This method has become particularly relevant with improvements in computational power and the availability of whole slide imaging scanners which might even replace the optical microscope as the primary tool in pathology [12].

The commonly used algorithms in the analysis of medical images include statistical (co-occurrence), structural (fractal), model-based (Markov random fields), and spectral (Gabor filters, wavelet transform, and curvelets). These were previously exploited for analysis of either unspecifically [13] or specifically [14,15] stained histopathology specimens, However, although they provide very good prognostic value, with AUCs reaching up to 0.77 [16], the obtained features are very abstract due to their complex calculation and thus unsuitable for straightforward identification of the structural prognostic clues. For this reason, our current study exploits a simple computational size and shape analysis of epithelial cell patches in breast tumors. The primary tumor is the valuable source of prognostic clues as the site of metastatic dissemination [17], while distant metastasis is the main cause of death and thus the most relevant event for prognostication of individual disease outcome in breast cancer. Epithelial cell structures were analyzed because this cell type is the origin of a neoplastic transformation in 99% of breast cancers. Such structures typically exert enormous variability in size and shape between different tumors and even within the same tumor. We thus hypothesized that the shapes and sizes of malignant clusters present the abundant source of prognostic information by reflecting the growth patterns of neoplastic cells which, in turn, might reveal the metastatic potential of a tumor.

While the prognostic value of epithelial cluster number, size, distribution, staining intensity, and texture within breast tumors [14,15,16,18,19] and the peritumoral buds [20] has been previously investigated, this is the first study aimed to comprehensively analyze the distribution of prognostic information among intratumoral malignant cell clusters according to their shape and size.

Based on the pressing need to improve breast cancer prognosis, this study explored the strategies to extract the maximum prognostic information by computational size and shape analysis of malignant cell clusters in breast tumors. A large number of cluster subsets were selected by filtering and subsequently prognostically evaluated by their size, shape, and count. Furthermore, we aimed to precisely identify the epithelial structure(s) with the highest content of prognostic information.

## 2. Results

### 2.1. Patient Characteristics

Selection of breast cancer patients was retrospective and based on the absence of systemic treatments with hormonal or cytotoxic drugs. This was according to recommendations for lower risk patients effective in the year 1993 for the smaller size tumors classified as pT1 and pT2, grade 1 and grade 2, and without lymph node involvement or metastasis (N0M0) breast carcinoma (Table 1). All patients received local treatment by surgery and radiation. In the studied patient group, metastases occurred in liver, lungs, bones, skin, and in muscle (Table 1). The median time to metastasis was 61 months, ranging between 16 and 155 months, while the median follow-up time for patients without metastasis was 147 months by a reverse Kaplan-Meier method, ranging between 77–165 months. Epidermal growth factor receptor 2 (*HER2*) status was positive in 22 patients (Table 1).

### 2.2. Prognostic Performance of the Clinicopathological Features

With TNM and histologic grade being ineffective in this early breast cancer patient group, only tumor size showed prognostically significant association, with an AUC of 0.65 and *p* = 0.04 (Table 2). It is of note that *HER2+* in Table 2 defines a positive *HER2* amplification, while *HER2-*enriched represents the estrogen receptor-negative (ER−), progesteron receptor-negative (PR−), *HER2+* molecular subtype of breast cancer [21]. All parameters in Table 2 were evaluated for prognostic performance by receiver operating characteristic ROC analysis and use of distant metastasis as the endpoint. In order to avoid the bias introduced by categorization of the measured values, the statistical analysis for age, tumor size, ER, and PR was performed by use of continuous values, without any cut-off categorization. Thereby, the age was measured in years, tumor size in millimeters, and estrogen/progesteron receptors in fmol/mg tumor tissue. Other parameters presented in Table 2 were intrinsically categorical. AUC values in the 0.0–0.5 range indicate an association with low risk and the 0.5–1.0 range with high metastasis risk. AUC values farther away from its random performance mid-point at 0.5 indicate an improved discrimination efficiency. Thereby, 0.3–0.4 and 0.5–0.6 are considered as fair discrimination performance, 0.2–0.3 and 0.7–0.8 as good, 0.1–0.2 and 0.8–0.9 as excellent, and 0.0–0.1 and 0.9–1.0 as almost perfect. The prognostic performance of the established clinicopathological features in Table 2 is presented for comparison with the newly discovered features presented in Table 3 and Table 4.

### 2.3. Optimization of the Image Binarization as the Strategy for Prognostic Performance Improvement

**Figure 1:**
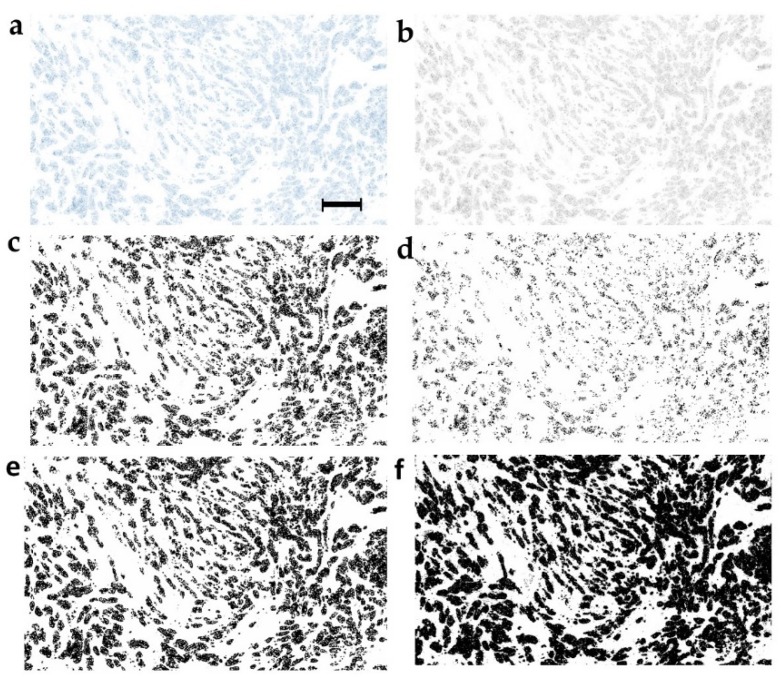
Optimization of the binarization threshold. (**a**) The blue channel of the representative pan-cytokeratin-stained tumor tissue section and its (**b**) 8-bit greyscale version. Binary images obtained from the 8-bit format by (**c**) automatic thresholding, (**d**) 220-threshold, (**e**) 240-threshold, and (**f**) 250-threshold. Scale bar: 300 μm.

The binarization step is obviously critical for the particle analysis because it is performed on binarized images. The circularity feature calculated in automatically thresholded images delivered an AUC of 0.67 and *p* = 0.02, thus prognostically exceeding the clinicopathological parameters (Table 2 and Table 3). The image format presented in Figure 1a presents an exemplary original blue pan-cytokeratin staining, while the 8-bit transformed image in Figure 1b contains 256 shades of grey ranging from 0 (black) to 255 (white). Figure 1c shows an image produced by automatic binarization of the 8-bit image. The manually set 240-threshold produced the binary image (Figure 1e) similar to that obtained by automatic thresholding (Figure 1c). For optimization, we also used the thresholds producing lighter (220) and darker (250) images (Figure 1d,f). Table 3 indicates that the 240-threshold provided the best prognostic value in terms of the number of prognostically significant features, however, the prognostic performance still did not exceed an AUC of 0.67/0.33, generally considered only as “fair” (Table 3).

### 2.4. Selection of Different Particle Subsets by the Circularity and Size Filtration

**Figure 2:**
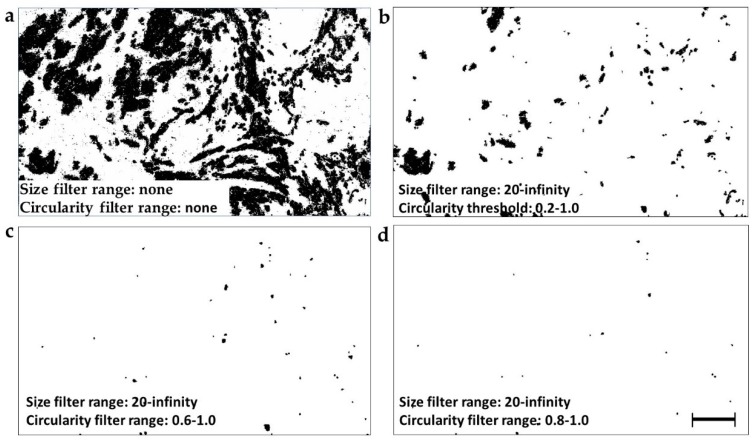
Particle filtering targets analysis to different particle subsets. (**a**) Representative unfiltered pan-cytokeratin histopathology staining, binarized as indicated in Figure 1f. Particle subsets were selected from this unfiltered image by: (**b**) the circularity filter set to 0.2–1.0 and the particle size filter set to 20-infinity, (**c**) circularity filter set to 0.6–1.0 and particle size filter at 20-infinity, (**d**) circularity filter set to 0.8–1.0 and particle size filter at 20-infinity. It is obvious that higher settings of the circularity filter selected the small and round particles resembling individual cells. Magnification 100×. Pixel size = 1.4 μm. Scale bar: 300 μm.

Particle analysis procedure initially outlines all particles in a binary image and then proceeds to their counting and measurement. The total number of analyzed particles (Figure 2a) can be narrowed by circularity and size filters (Figure 2b–d). Such filtering is based on the fact that particles with shapes (circularity) and sizes falling outside of the filter range are ignored. A total of 80 particle subsets were produced by combination of 4 binarization thresholds (automatic, 220, 240, 250), 5 circularity thresholds (0, 0.2; 0.4; 0.6; 0.8), and 4 object size thresholds (10, 20, 50, 100 pixels). Figure 2 illustrates the vast impact of the circularity filter on the count, size, and shape of particles selected for analysis. The circularity filter was thus the main selection tool and the applied circularity thresholds of 0, 0.2, 0.4, 0.6, and 0.8 were chosen to cover the entire theoretical circularity range from 0–1.0. Circularity settings above 0.8 were not used due to zero particles selected in most images. Without any filtering, the exemplary histopathological image in Figure 2a provided 170 particles. The circularity filter setting at 0.2–1.0 has narrowed the selected particle subset to 146 (Figure 2b), while the circularity range at 0.6–1.0 included 45 particles (Figure 2c) and circularity at 0.8–1.0 only 20 particles (Figure 2d). The average particle size also declined from 2331 to 260, 64, and 46 pixels, respectively. The average Feret maximum diameter has similarly decreased from 46 to 21, 11, and 9 pixels, corresponding to 64, 29, 15, and 13 µm. The particle diameters selected by the highest 0.8–1.0 circularity filter, were within the size range of an individual cell (9.4–21.4 μm), while 85% of particles were within 9–15 μm range (Figure 2d).

### 2.5. Identification of the Prognostically Optimal Particle Subset and the Consisting Particles

**Figure 3:**
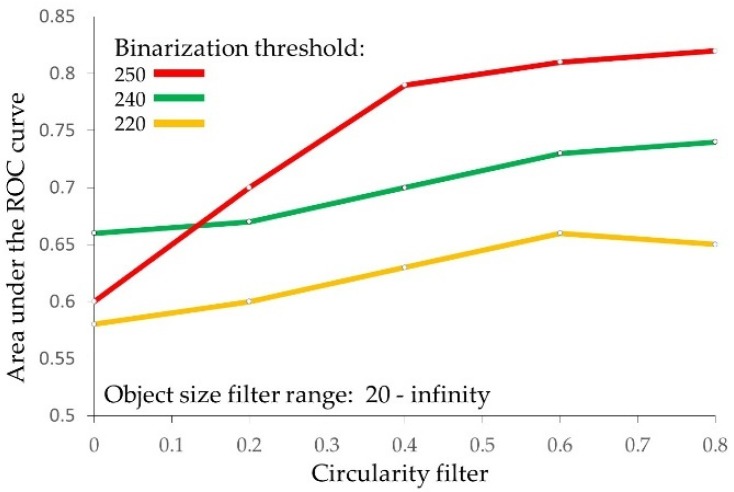
Circularity filter affects the prognostic performance of the particle count feature. The circularity threshold increases from 0.0–0.8 resulted in consistent incremental improvement of the prognostic performance for the particle count feature. The particle size filter setting was constant at 20-infinity pixels. This figure also demonstrates how the prognostic performance of the particle count feature depends on the binarization threshold.

By evaluation of all 80 particle subsets defined by particle size and shape filters, we identified the prognostically optimal subset, specified by the 0.8–1.0 circularity filter and the 20-infinity object size filter settings, while binarization was performed by the 250-threshold (Table 4, Figure 3). This subset has accomplished a remarkable level of prognostic performance, with the particle *count* feature associating with metastasis outcome by an AUC of 0.82 (Table 4). Consistently, the rate of metastasis in patients with a high count of these particles was much higher in comparison to the low count patient subgroup (Table 1). In this optimal particle subset, all features provided AUC values above 0.5, indicating their association with the increased risk of metastasis (Table 3). Interestingly, while binarization with the 240-threshold was prognostically optimal for particles prior to their filtering, the 250-threshold provided the best prognostic performance upon particle filtering, followed by the 240- and 220-thresholds (Figure 3 and Table 3 and Table 4). It is important to note that the prognostic evaluation presented in Table 2, Table 3, Table 4 and Table 5 was performed by use of all 20 distant metastasis occurrences as events. When the prognostic evaluation was performed separately by metastasis location: 3 in the liver, 8 in lung, and 7 in bones, the prognostic significance was achieved by the count (AUC = 0.76/*p* = 0.03) and the total area (AUC = 0.77/*p* = 0.02) features for lung metastases. The average size feature associated with bone metastases by AUC = 0.74/*p* = 0.03. These results are not presented in Table 4 because the number of metastases in each location was below the 17 events required by the sample size calculation.

**Figure 4:**
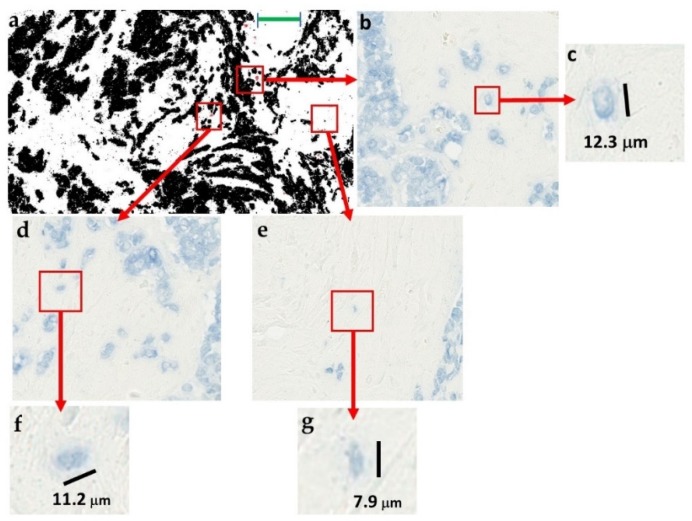
Identification of the structural prognostic clues in breast tumor histopathological specimens stained for epithelial cells. (**a**) The exemplary pan-cytokeratin stained tumor tissue section is identical as in Figure 1f and Figure 2a to facilitate comparison. Particles filtered by the ranges of circularity = 0.8–1.0 and particle size = 20-infinity are indicated by overlay masks in red. (**b**,**d**,**e**) Enlargements show their small size and stromal localization, detached from large epithelial cell clumps. (**c**,**f**,**g**) Maximum enlargements show that the selected particles are recognizable as scattered individual epithelial cells by their morphology, size, and pan-cytokeratin immunostaining. Scale bar for (**a**): 300 μm.

**Figure 5:**
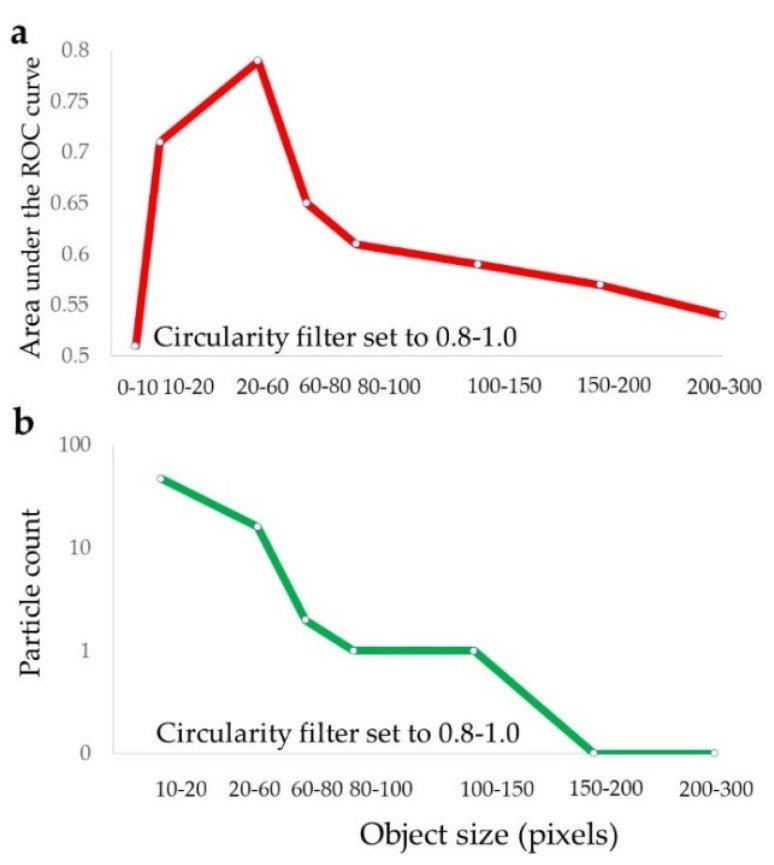
The particle size distribution in relation to prognostic performance and particle count. (**a**) Prognostic performance of the particle count feature is dependent on particle size. The maximum prognostic AUC of 0.80 was obtained by the particle size range of 20–60 pixels (approx. 7–11 μm in maximum diameter). This peak corresponds to the size of an individual cell. (**b**) The size distribution of particles. Smaller particles were more abundant. Forty-seven particles were counted in the 10–20 pixel size range, 16 in the prognostically optimal 20–60 pixel size range, and only 2 in the larger range of 60–80 pixels. For easy comparison, all data refer to the identical exemplary image as shown in Figure 2a and Figure 4a.

Identification of the prognostically relevant particles was based on matching between particles selected in binary images (Figure 4a) and their corresponding original color format (Figure 4b,d,e). Figure 4a indicates the selected particles as red outline masks, while the magnified color panels (Figure 4b,d,e) show morphological detail and stromal localization of these particles, in between the large epithelial cell clusters. The size of these particles, together with their circular shapes, pan-cytokeratin immunoreactivity and visual inspection under maximum magnification (Figure 4c,f,g), supported their identification as scattered individual epithelial cells. This result was supported by the finding that particles in the single cell size range (20–60 pixels, approx. corresponding to a diameter range of 7–11 μm), provided the best prognostic performance (Figure 5a). Furthermore, the number of particles larger than a single cell was very small in this prognostically optimal subset (Figure 5b). The size filter thus served the purpose to define the range of particle sizes providing the best prognostic value (Figure 5a) and also to exclude the noise from very small particles ranging from 1–10 pixels (Figure 5a) which did not contain any prognostic information.

### 2.6. Multivariate Analysis of the Clinicopathological and Particle Analysis Features

Multivariate analysis (Table 5) included all the clinicopathological and particle analysis features satisfying the selection entry criterion of *p* ≤ 0.2 obtained in univariate analysis presented in Table 2 and Table 4. The variables thus included were: age, grade, ER, particle count, average size, total area, and circularity. Variables were removed using backward elimination according to the selection stay criterion of *p* < 0.05. Table 5 presents only the remaining variables, indicating the independent prognostic value for the count feature (Table 5).

## 3. Discussion

We report the first exhaustive and hypothesis-free prognostic screening of the epithelial cell clusters in breast carcinoma by their size and shape. Epithelial cells are of particular prognostic relevance because the vast majority of breast cancers develop by their neoplastic transformation.

This study did not analyze predefined morphological features but focused on the identification of novel prognostic clues. Our computational analysis was therefore not an attempt at the automation of the visual microscopic analysis, but rather a discovery tool which enabled comprehensive morphological evaluation and classification into size and shape subsets of the hundreds and often thousands of epithelial clusters that are found in a typical microscopic field of view. Malignant epithelial cells typically occur in breast tumors as irregular clusters which reflect their growth patterns. We hypothesized that such clusters present the abundant source of novel prognostic clues because of their wide variability in size and shape distribution among different tumors. These clusters were clearly defined by immunostaining and could thus be easily outlined and analyzed by ImageJ software.

Previous studies aimed at the identification of novel structural prognostic clues in tumor specimens have mostly focused on tissue sections unspecifically stained by hematoxylin and eosin. This type of staining is by far more complex than immunostaining for epithelial cells and therefore required a rather elaborate methodological approach [22]. The identified prognostic clues were inevitably also complex, therefore only usable by computational analysis. Yet, the sporadic individual epithelial cells identified as the prognostic clue by our approach are structurally so simple and consistent that they can be quantified and verified even by standard microscopy.

The prognostic AUC of 0.35 obtained in this study for the unfiltered total pan-cytokeratin stained area was in line with the obtained AUC value of 0.30 previously reported for the similar pan-cytokeratin staining intensity feature [14]. Both features thus consistently associated with the low metastasis risk. However, upon particle selection by the circularity filter, the reversal of the prognostic direction for the total pan-cytokeratin stained area was notable from its AUC of 0.35 to 0.78. This discrepancy can be explained by the small overlap of unfiltered particles and the particle subset selected by the circularity threshold of 0.8. The overlap was only 0.21%, thus allowing for the opposite prognostic performance of the two particle subsets.

The previous study by Wang et al. of pan-cytokeratin stained breast tumor histopathology reported the best prognostic AUC of 0.66 for circularity [19]. This result was nearly identical to our initial data obtained prior to the prognostic enhancement achieved by particle filtering. The circularity of malignant cell clusters is a geometrical and mathematical feature with a downside that it can only be quantified by computational image analysis. However, the above data provided an indication that increasingly circular particles might deliver much stronger association with high metastasis risk. By following this lead, we extended our research effort further from the cluster circularity feature, also previously reported by Wang et al. [19], in order to discover a prognostic marker with a better prognostic performance that can also be easily visually quantified. This was a major achievement in view of the fact that computer-based imaging studies rarely manage or even aim to precisely identify simple prognostic clues. By the reported use of shape and size filters, we managed to extract the particles with the prognostic value by AUC = 0.82 and hazard ratio (HR) of 17.2, exceeding that reported (AUC = 0.66, HR = 1.45) by Wang et al. [19]. The stromal localization of these scattered individual epithelial cells, in between the large epithelial cell clusters, indicated their malignant nature because normal epithelial cells in the breast form large structures with defined shapes and cannot be found as detached in tumor stroma. The sporadic individual epithelial cells identified here as the best prognostic performers are comparable to the previously reported tumor buds described as small clusters of 1–5 epithelial cells that in 3D reconstruction appear as an initial step of detachment from the main tumor mass into a stroma, ahead of the invasive front of the tumor [23]. Tumor budding was mostly investigated in colorectal carcinoma and it is believed to be closely related to the epithelial–mesenchymal transition and represents the first step of migration, invasion, and metastasis [20,24]. In agreement with our current results, such tumor buds were reported as markers of the adverse clinical outcome [20]. However, the important distinction is the size, as tumor buds range between 1–5 cells, while the prognostic marker identified in our current study is narrower in size range, representing a single cell. Furthermore, the prognostic value of intratumoral epithelial single cells described in this study is much higher by HR = 14.8 in comparison to the best previously reported for the peritumoral buds achieving an HR of 6.5 [20]. Another distinction is the location because the tumor buds were primarily investigated in the peritumoral area, while we analyzed the intratumoral epithelial structures. The single study describing intratumoral buds showed a significant association with tumor grade and ER positivity [25]. However, due to the short follow up, the prognostic performance was evaluated only on the basis of association with these pathological parameters, while the more reliable prognostic events such as distant metastasis occurrence were not available [25], thus restricting any prognostic performance comparison with our current results.

The circulating tumor cells (CTC) have received much attention [26] as their content is known to correlate with metastasis occurrence by an AUC of up to 0.73 [27]. Intriguingly, we report here a stronger association with metastasis occurrence for the malignant cell count in tumor stroma, even prior to their dissemination into the blood. Superior prognostic performance of these stromal epithelial cells might be explained by the ease and reliability of their quantification in tissue sections by computational analysis, while CTC quantification in the blood is far more challenging and possibly inaccurate [28].

Advantages of this study include the identification of the leading prognostic histomorphological clue that can be easily quantified by visual microscopy without any need for specialized software. Computer-based imaging studies exceptionally rarely manage or even aim to identify simple prognostic clues. The achieved excellent prognostic performance is another major benefit of the current study. Advantages of this study further include the use of whole-slide images instead of the more common tissue microarrays which are by far smaller. Computational analysis has also enabled an automatic size and shape filtering and automatic quantification of malignant clusters according to their size and shape. Furthermore, the used patient group did not include any systemic treatment which could interfere with metastasis occurrence. We needed to retrieve the 26-year-old archived samples to assemble such a group, as more recent treatment protocols prescribe systemic cytotoxic and/or hormonal treatments to most patients. To assess the robustness of the prognostic evaluation, we performed a bootstrap validation as a bias-correction method for the ROC and Cox regressions. Advantages of the study design further include the twofold evaluation of the prognostic significance, by ROC and Cox regression analyses, followed by the Cox multivariate analysis as the estimation of potential clinical usefulness. The convenience of the Cox regression was in its consideration of the time to metastasis while ROC analysis only accounts for metastasis outcome. However, the downside of Cox regression is its requirement for categorized data which introduces bias into the prognostic evaluation. Therefore, we also included ROC analysis which is commonly used in prognostic performance evaluation but makes use of continuous data values. Due to the long term follow up, this study was able to accumulate a sufficient number of distant metastasis events to perform a prognostic evaluation. This presents the major advantage as distant metastasis is by far the most reliable event for prognostication of breast cancer outcome, based on the fact that it causes 90% of cancer deaths.

Although it exceeded the requirement estimated by the prospective sample size analysis, the group of 102 patients is a limitation of this study. However, the systemically untreated patient group and the bootstrap validation supported the reliability of the obtained results. The prognostic validation is widely understood as the generalizability test which can be performed by internal validation within the existing patient group and/or the external validation in another unrelated patient group. The current study has performed the prognostic validation by the internal bootstrap bias-correction method [29]. Additional validation by studies in an extended patient group and external groups would be needed to further characterize the prognostic clinical validity of the analysis performed in this study. Furthermore, although the employed computational analysis technique is fully objective, the overall workflow still included residual subjectivity at the level of selection of representative tumor histopathology areas for analysis. The retrospective design of the prognostic model was another limitation. Besides, pan-cytokeratin AE1/AE3 antibody cocktail immunostains both normal and malignant breast epithelial cells. This limitation was largely overcome by the selection of the predominantly malignant tumor areas, based on morphological criteria. Therefore, the pan-cytokeratin staining in the current study indicated the growth patterns of malignant cells.

## 4. Materials and Methods

Writing of this report was done to include all relevant experimental detail according to recommendations for tumor marker prognostic studies [30].

### 4.1. Ethics Approval Statement

The study was approved by the Institutional Review Board (Belgrade University, School of Medicine, approval #29/VI-4) and conforms with The Code of Ethics of the World Medical Association (Declaration of Helsinki), printed in the British Medical Journal (July 18, 1964) and its 7th revision in 2013.

### 4.2. Patient Group

Patient data were obtained in a de-identified form without identifiers that could enable re-identification (Safe-Harbour methodology of the 2012 Health Insurance Portability and Accountability Act). All patients were female Caucasian, treated in the same year (1993) at the Institute of Oncology and Radiology of Serbia. Sixty-nine percent of patients were positive for estrogen receptor (ER, median of 32 fmol/mL) and 24% were positive for progesterone receptor with a median of 6 fmol/mL, based on the respective cutpoints of 10 fmol/mg and 20 fmol/mg. Estrogen and progesterone receptors were measured by dextran-coated charcoal assay [31]. The median age at diagnosis was 57 years (range 37–80). The prospective sample size calculation was based on a pilot study including 40 patients and required 85 patients with 17 positive cases for alpha = 0.05, beta = 0.20, and AUC effect size of 0.72/0.28 (MedCalc Software, Ostend, Belgium). The actually obtained best AUC was 0.82, with a sample size of 102 patients of which 20 cases were metastasis positive.

### 4.3. HER2 Amplification Testing

The evaluation of *HER2* amplification was not performed at the time of diagnosis in year 1993 because it was not a part of routine clinical testing at that time. This test was performed in 2012 by the SPOT-Light^®^ HER2 CISH Kit, (Cat. #84-0150, Zymed/Invitrogen, Thermo Fisher Scientific Corp. Waltham, MA, USA), based on chromogenic in situ hybridization (CISH), as we previously described in full detail [32]. This kit uses digoxigenin-labelled DNA probes to quantitatively determine *HER2* amplification in formalin-fixed, paraffin-embedded breast carcinoma tissue sections. Hybridization results were evaluated in the 400× and 1000× magnification fields by a brightfield Olympus BX51 microscope. One to five gene copies per nucleus were defined as no amplification, while more than 6 gene copies per nucleus defined a positive amplification. We have previously reported a high agreement between the immunohistochemistry and CISH methods in the evaluation of *HER2* gene amplification [32,33].

### 4.4. Study Design

Histopathology images from 102 patients were stained by pan-cytokeratin antibody to label epithelial cell clusters which could be outlined by ImageJ software. The circularity filter was used to select a total of 80 subsets of epithelial clusters differing in shape and size. These subsets were further screened for their prognostic value and the particles in the optimal subset identified as scattered individual epithelial cells.

### 4.5. Image Analysis Workflow

The workflow included immunostaining, selection of tissue sections, image acquisition, stain decomposition, binarization, particle analysis, and prognostic evaluation and validation. These steps are described below.

### 4.6. Immunostaining

Tissue was obtained during surgical removal of a tumor. Primary breast tumor tissue was formalin-fixed, paraffin-embedded, and cut to produce 4 μm whole sections. A heat-mediated antigen retrieval was done in a water bath set to 95 °C for 40 min in EDTA pH 8 buffer. Endogenous peroxidase was quenched with 3% H_2_O_2_ in methanol for 30 min and 5% goat serum was used for preincubation. The whole tissue sections were incubated with the CD8 monoclonal rabbit antibody (ThermoFisher Scientific, Waltham, MA, USA; #RM-9116-S1), followed by the pan-cytokeratin primary antibody clones mAE1/AE3 (Dako, Glostrup, Denmark, #M3515) in 5% goat serum for 60 min. This antibody cocktail stains epithelial cells by detecting cytokeratins 1–8, 10, 14–16, and 19. Washing was performed in PBS and the secondary goat anti-rabbit IgG HRP conjugate added (Jackson ImmunoResearch Laboratories, West Grove, PA, USA; #111-035-144), followed by alkaline phosphatase conjugated polyclonal goat anti-mouse IgG (Southern Biotech, Birmingham, AL, USA; #1030-04) in 5% goat serum. Chromogens were nickel-enhanced DAB (Vector Laboratories, Burlingame, CA, USA) and subsequently the FastBlue RR (Sigma-Aldrich, St. Louis, MO, USA). Counterstain was not performed in order to highlight only epithelial cells.

### 4.7. Selection of Tissue Sections

To achieve maximal reproducibility and validity, the pathologist (KK) selected the tissue sections containing the growth patterns characteristic for each individual tumor, with the highest content of pan-cytokeratin stained malignant cells and without artefacts. Pan-cytokeratin-stained cell arrangements were identified as normal or malignant according to their morphology.

### 4.8. Image Acquisition

Color images were acquired by use of the Hamamatsu-XRC12000 NanoZoomer high-resolution digital slide scanner (Hamamatsu City, Japan).

### 4.9. Stain Decomposition

Blue (pan-cytokeratin) and brown (CD8) channels were decomposed as previously described in detail by Li and Plataniotis [34]. All downstream image analysis in this study was performed in the blue pan-cytokeratin channel.

### 4.10. Image Binarization

Blue images were transformed to the 8-bit greyscale format by the run (“8-bit”) command of the Fiji/ImageJ version 1.52n, an open image analysis software [35]. Images were further transformed to a binary format by the run (“Make Binary”) command for automatic thresholding or setThreshold(0, 220), setThreshold(0, 240), and setThreshold(0, 250) commands for fixed thresholding. Automatic thresholding applies a different threshold to each image based on the tonal distribution histogram, while set thresholding applies a fixed threshold to each image in the batch.

### 4.11. Image Analysis

A total of 532 images provided approximately 5 representative images per patient, for 102 patients. The binarized images were analyzed by the “analyze particles” function in ImageJ. This analysis performs an automatic segmentation which distinguishes particles from their background and outlines the individual particles. Particles were subsequently counted, and their size and shape defined by parameters such as average size in pixels, maximum Feret diameter, perimeter, circularity, and solidity. The first three parameters are size descriptors, while circularity is a shape descriptor and solidity a density descriptor. Solidity is calculated as the area of a particle divided by its convex hull area, whereby a solid object has a value of 1, while an object with irregular boundary or holes has a value of less than 1. Circularity or roundness is calculated as 4π × area/perimeter^2^. A circularity value of 1.0 indicates a perfect circle, while values approaching 0.0 reflect an increasingly elongated shape.

### 4.12. Prognostic Evaluation

Values of the above-mentioned features were averaged among 5–6 images available for each patient, followed by the prognostic evaluation by ROC and Cox regression analyses, with metastasis occurrence as the endpoint event. These tests compare the prognosticated and actual metastasis outcomes. The area under the rate of change curve (AUC) is a quantitative method commonly used to assess efficiency of discrimination with a binary endpoint. Discrimination is the capability of prognostic features to stratify patients with and without the actual metastasis occurrence. AUC = 0.5 represents chance discrimination, while perfect discrimination equals 0.0 or 1.0. AUC is calculated by use of continuous feature values, while Cox proportional hazards regression analysis is calculated by use of categorized data. Data was categorized by dividing patients into low- and high-risk subgroups with an optimal cutpoint selected by X-tile 3.6.1 software (Yale University, New Haven, CT, USA). Each feature satisfied the proportional hazards assumption based on the Schoenfeld residuals by phtest (Stata/MP 13 package, StataCorp, College Station, TX, USA). The hazard ratio (HR) is the effect size of the Cox regression reflecting the metastasis rates in high- and low-risk patient subgroups. It indicates chance performance at HR = 1.0. As for AUC, the HR values above its chance performance indicate an association with high risk, while HR values under 1.0 point to low risk markers. The independence of each prognostic factor was tested by multivariate Cox proportional hazards regression analysis. ROC analysis and Cox regression can disagree in their prognostic evaluation because the data categorization step might introduce bias. Furthermore, Cox regression takes the time interval from surgery to metastasis into account, while ROC analysis does not.

### 4.13. Validation

The over-optimism of the ROC (Stata/MP 13) and Cox (IBM SPSS) analysis was corrected by the bootstrap internal validation with 1000 data resamples [36].

## 5. Conclusions

Our findings show that malignant cell clusters in breast tumors provide prognostic information by their count, size, and shape. We also report, for the first time, that the smallest of these patches provide a particularly high performance in prognostication of metastasis occurrence and can be identified as scattered individual epithelial cells in tumor stroma. Their count was characterized as an independent prognostic factor. Internal validation performed by bootstrap suggests that the model is generalizable. Scattered individual epithelial cells are easy to implement as markers of high metastasis risk because they can be reliably and widely identified, quantified, and investigated either by computational analysis or visual microscopy without the use of any specialized software. The clinical relevance of prognostic improvement is based on its role in early individual treatment decisions which affect the quality of life and survival.

## Figures and Tables

**Table 1:** Patient characteristics ^a^.

Parameter	*n*	Metastasis (%) ^b^
*HER2* status		
*HER2−*	80	21
*HER2+*	22	20
ER status (cut off = 20 fmol/mg)		
ER−	32	13
ER+	70	23
PR status (cut off = 10 fmol/mg)		
PR−	64	25
PR+	38	17
Tumor size (cm)		
≤ 2	73	12
2–5	26	37
≥ 5	2	50
Nodal status		
N0	102	20
N+	0	0
Histologic grade		
G1	9	33
G2	92	17
G3	1	0
Count of individual scattered epithelial cells ^c^		
Low count (0–7.6)	60	5
High count (8.3–37.0)	42	40
Metastasis		
lungs	8	100
bones	7	100
liver	3	100
skin	1	100
muscle	1	100
none	82	0

^a^ The total number of patients was 102. ^b^ Percent of actual metastasis occurrence in each patient subgroup. ^c^ The average counts per patient were divided into the low count and high count subgroups by the optimal cut off. Abbreviations: HER2, epidermal growth factor receptor 2; ER, estrogen receptor; PR, progesterone receptor.

**Table 2:** Prognostic significance of the clinicopathological features ^a^.

Parameter	AUC	*p*-Value	95% CI
Age	0.60	0.15	0.48–0.73
Tumor size	0.65	0.04 *	0.51–0.78
Grade	0.45	0.50	0.31–0.60
ER	0.60	0.14	0.46–0.75
PR	0.48	0.80	0.33–0.63
HER2+	0.49	0.45	0.32–0.67
HER2-enriched	0.47	0.67	0.33–0.61
Triple negative	0.46	0.55	0.32–0.60

The receiver operating characteristic (ROC) curve analysis was used for evaluation of prognostic significance. ^a^ bootstrap corrected * *p* ≤ 0.05. Abbreviations: AUC = area under the ROC curve; CI = confidence interval; HER2 = human epidermal growth factor receptor 2; HER2-enriched = ER−, PR−, HER2+; Triple negative = ER−, PR−, HER2−; CI, confidence interval.

**Table 3:** Prognostic optimization by variation of the binarization thresholds ^a^.

Parameter	AUC/95% CI ^b^/*p*-Value ^b^			
	Binarization: auto Circularity: 0.0–1.0 Size: 20-infinity	Binarization: 220 Circularity: 0.0–1.0 Size: 20-infinity	Binarization: 240 Circularity: 0.0–1.0 Size: 20-infinity	Binarization: 250 Circularity: 0.0–1.0 Size: 20-infinity
Count	0.57 0.46–0.71 0.31	0.58 0.44–0.71 0.29	0.66 0.53–0.80 0.03 *	0.60 0.47–0.73 0.18
Total area	0.37 0.25–0.50 0.08	0.35 0.22–0.49 0.04 *	0.35 0.24–0.47 0.04 *	0.54 0.39–0.68 0.63
Average size	0.40 0.27–0.53 0.15	0.37 0.23–0.50 0.06	0.33 0.21–0.46 0.02 *	0.44 0.29–0.59 0.41
Circularity	0.67 0.55–0.78 0.02 *	0.59 0.45–0.73 0.21	0.65 0.59–0.81 0.04 *	0.62 0.50–0.75 0.09
Solidity	0.62 0.50–0.73 0.11	0.54 0.39–0.68 0.61	0.57 0.43–0.70 0.37	0.57 0.43–0.71 0.32

^a^ ROC analysis was used for evaluation of the prognostic performance by use of continuous data. ^b^ corrected by bootstrap. * *p* ≤ 0.05.

**Table 4:** Prognostic significance of the particle analysis features obtained by the optimal settings of particle filters and binarization.

Parameter	AUC ^a^	95% CI	*p*-Value	HR ^b^	95% CI	*p*-Value
Count	0.82	0.72–0.90	0.000 *	14.8	5.3–242	0.001 *
Total area	0.77	0.68–0.86	0.000 *	17.2	5.7–230	0.001 *
Average size	0.63	0.54–0.77	0.03 *	11.9	2.1–28.8	0.001 *
Circularity	0.62	0.51–0.74	0.09	12.8	3.3–61.6	0.02 *

^a^ ROC analysis was used for prognostic evaluation by use of continuous data. The prognostic performance is shown only for the optimal particle subset which was selected by the circularity filter range 0.8–1.0 and object size filter range 20-infinity, while binarization threshold was 250. Corrected by bootstrap. ^b^ Cox proportional hazards regression analysis was used for prognostic evaluation by use of data categorized by an optimal threshold. The prognostic performance is shown only for the optimal particle subset. Corrected by bootstrap. * *p* ≤ 0.05. Abbreviations: HR = hazard ratio.

**Table 5:** Multivariate Cox regression analysis of the clinicopathological and particle analysis prognostic features ^a^.

Feature	*p*-Value ^a^	HR	95%CI ^a^
Age	0.03	5.5	1.4–1264869
Count	0.001	16.1	5.7–344551

Multivariate stepwise regression analysis was performed by inclusion of the clinicopathological and particle analysis features to capture the prognostic redundancy. The entry criterion was *p* ≤ 0.2 and the remain criterion *p* ≤ 0.05. ^a^ bootstrap corrected.
